# Ultra-High Molecular Weight Polyethylene/Titanium-Hybrid Implant for Bone-Defect Replacement

**DOI:** 10.3390/ma13133010

**Published:** 2020-07-06

**Authors:** Aleksey V. Maksimkin, Fedor S. Senatov, Kirill Niaza, Tarek Dayyoub, Sergey D. Kaloshkin

**Affiliations:** 1 Laboratory of Hybrid Nanostructured Materials, National University of Science and Technology “MISIS”, 119049 Moscow, Russia; 2 Center of Composite Materials, National University of Science and Technology “MISIS”, 119049 Moscow, Russia; Senatov@misis.ru (F.S.S.); mobiad@yandex.ru (K.N.); tarekzd@windowslive.com (T.D.); kaloshkin@misis.ru (S.D.K.)

**Keywords:** polymer implant, UHMWPE, titanium alloy, mechanical properties, porous, bone

## Abstract

A hybrid implant with a structure mimicking that of natural bone was developed. Titanium alloy Ti–6Al–4V prepared with three-dimensional (3D)-printing technology was used to simulate the cortical-bone layer. The mismatch in the mechanical properties of bone and titanium alloy was solved by creating special perforations in the titanium’s surface. Porous ultra-high molecular weight polyethylene (UHMWPE) with high osteogenous properties was used to simulate the cancellous-bone tissue. A method for creating a porous UHMWPE structure inside the titanium reinforcement is proposed. The porous UHMWPE was studied with scanning electron microscope (SEM) to confirm that the pores that formed were open, interconnected, and between 50 and 850 μm in size. Mechanical-compression tests done on the obtained UHMWPE/titanium-hybrid-implant samples showed that their mechanical properties simulated those of natural bone.

## 1. Introduction

Reconstructing the structural integrity of damaged bone-tissue sections is currently considered a major problem. Commonly used materials for making bone implants are metals and their alloys, ceramics, and various kinds of polymers; each has its pros and cons. Metals and their alloys have good strength, excellent resistance to fatigue, and high ductility, which allow them to be used as a replacement for highly loaded bone tissue. However, their high elastic modulus may lead to stress shielding as a result of bone-tissue resorption [1]. Additionally, the metals’ tendency to significantly corrode reduces their biocompatibility, and corrosion products can poison the surrounding tissue [2,3].

Ceramic materials have high biocompatibility and bioactive properties, high strength, and high resistance to corrosion and wear [4]. However, ceramics have low resistance to fatigue, which makes these materials rather brittle [5]. Because of this, ceramic implants are considered to have a high risk of damage caused by impact stress. Moreover, as with metallic materials, ceramics’ high elastic modulus can lead to stress shielding.

Polymers have high biocompatibility, excellent resistance to corrosion, high ductility, and a modulus similar to that of natural bone [6,7]. They have great potential to be prepared as three-dimensional (3D) scaffolds that provide support for bone-cell and tissue growth [8,9,10]. However, most polymeric materials do not have sufficient strength and tend to creep. Additionally, they may degrade over time. Therefore, polymer implants cannot be used to replace large and highly loaded areas of bone tissue.

Successful bone-defect repair means that an implant should have a structure that mimics that of the bone being restored. Bone consists of two basic layers: cortical and cancellous tissue [11]. Cortical tissue contributes to reinforcing a bone’s mechanical characteristics. Cancellous tissue has osteogenic and osteoinductive properties that support cells and create optimal conditions for their growth.

**Figure 1:**
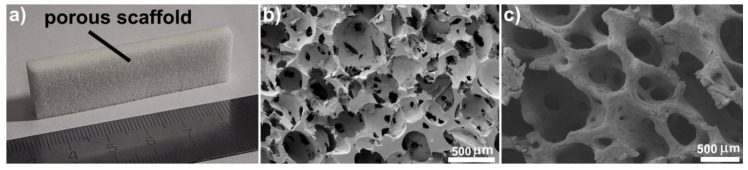
(**a**) Porous ultra-high molecular weight polyethylene (UHMWPE) scaffold; (**b**) SEM images of porous UHMWPE scaffold and (**c**) natural cancellous tissue.

Ultra-high molecular weight polyethylene (UHMWPE) is a widely used polymer in medical applications because of its high chemical resistance, biocompatibility, and mechanical and tribological properties. UHMWPE is used in implants for hip and shoulder arthroplasties, knee-joint replacements [12,13], and in spinal-disk prostheses [14]. Porous UHMWPE has good potential for use as a 3D porous scaffold in bone-defect replacement applications. A porous UHMWPE scaffold has a structure mimicking that of cancellous tissue, as shown in Figure 1, and it provides an ideal environment for cell growth [15,16]. The average pore size in porous UHMWPE corresponds to that of the cancellous tissue (100–600 microns [17,18]). The porous UHMWPE has open and interconnected pores, and its total porosity is close to 80%. Creating the porous UHMWPE scaffold using the salt-leaching method allows pore size to be easily changed to match that of the replaced bone area.

In medical applications, titanium alloys are commonly used in orthopedic and dental-implant products because of their reliable mechanical performance that allows them to replace hard bone tissue [19]. A lack of osseointegration into the bone is the main reason for the failure of titanium implants [20]. In 60% to 70% of clinical cases, titanium implants are revised because of aseptic loosening [20]. Strong fixation is necessary to guarantee the successful use of titanium implants.

Many investigations were carried out with the aim of reducing the elastic modulus of titanium-alloy implants. It is well known that the modulus of second-generation β–Ti alloys (80 GPa) is lower than that of first-generation orthopedic α+β-titanium alloys Ti–6Al–4V (110 GPa) and SUS 316L stainless steel (200 GPa) [21,22]. In addition, by changing the composition and preparation conditions of β–Ti alloys, the elastic modulus can be reduced to about 40 GPa [23,24,25]. However, β–Ti alloys may release some elements (e.g., Nb, Ta, and Zr) that can affect the surrounding tissue, and this may lead to a decrease in their biocompatibility compared to that of pure Ti and α+β–Ti alloys [26]. Another way to obtain a low modulus is by preparing a porous metal implant by using 3D-printing technology [27]. Depending on the type of used rapid-prototyping techniques and the process parameters, the obtained samples have a low elastic modulus ranging from 0.86 to 60 GPa [28,29].

In this study, a UHMWPE/titanium-hybrid implant with a structure mimicking that of natural bone was developed and explored.

## 2. Materials and Methods

### 2.1. Description of UHMWPE/Titanium-Hybrid Model

**Figure 2:**
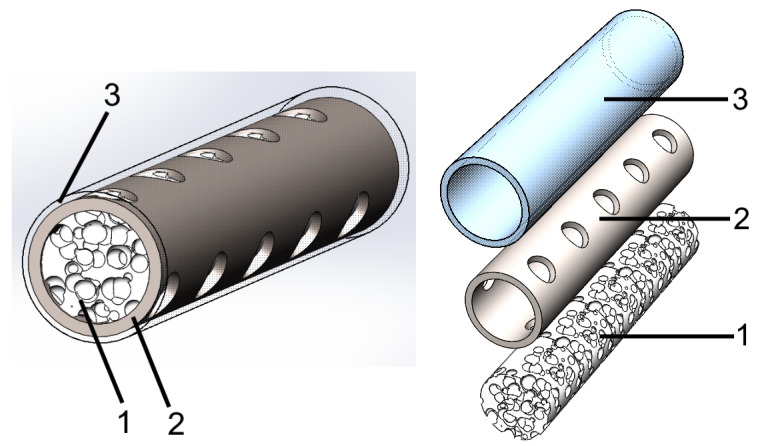
Hybrid-implant model with structure mimicking that of natural bone. (1) Porous UHMWPE with high osteoconductive properties (cancellous-tissue simulation); (2) titanium reinforcement with mechanical properties that were sufficiently close to those of natural bone (cortical-tissue simulation); (3) thin solid UHMWPE layer that created a smooth surface.

Figure 2 shows the model of the hybrid implant. The hybrid implant consisted of two main layers. One mimicked the cancellous tissue, as shown in Figure 2(1), and the other mimicked the cortical tissue of natural bone, as seen in Figure 2(2). To simulate the cancellous tissue, a porous UHMWPE scaffold was chosen to support bone-cell and tissue growth [15,16].

Titanium was chosen to simulate cortical tissue. The mismatch in the mechanical properties of bone and titanium was solved by creating special perforations in the titanium reinforcement’s surface. In the proposed hybrid-implant model, the main role of the metal part was to ensure that the implant had the optimal mechanical properties that it needed, while the polymer part provided the needed environment for new bone-cell growth.

The cortical tissue of natural bone has a smooth solid surface. This smooth surface is needed to prevent damage to the adjacent tissue (i.e., muscles) during movement. In order to create this smooth surface on the hybrid implant, a thin solid UHMWPE layer is proposed, as shown in Figure 2(3).

### 2.2. Three-Dimensional Printing of Titanium Reinforcement

Commercial spherical Ti–6Al–4V powder with a normal distribution size ranging from 20 to 63 µm was selected to manufacture titanium-reinforcement samples; average particle size was 48 microns.

Titanium reinforcements were obtained using selective-laser-melting (SLM) equipment (SLM 280 2.0, SLM Solutions Group AG, Lübeck, Germany) in an argon atmosphere to avoid any possible oxygen contamination. Deposition was performed at a laser power of 275 W, and scanning velocity was 1100 mm/s. Layer thickness was set to 30 μm, and track distance was 0.12 mm. Solid-titanium samples in the form of cylindrical tubes with a diameter of 10 mm, a height of 20 mm, and a wall thickness of 1 mm were obtained using SLM technology. The compressive mechanical properties of these types of titanium samples were used to construct a material model in SOLIDWORKS Simulation 2018 SP3 (Dassault Systèmes, Vélizy-Villacoublay, France).

### 2.3. Three-Dimensional Structural Model of Titanium Reinforcement with Reduced Elastic Modulus

The solid-titanium samples prepared by 3D printing had a rather high elastic modulus (116 GPa), as can be seen in Table 1. The elastic modulus of natural bone was in the range of 7–30 GPa [30,31,32]. This large difference between the elastic moduli of the artificial implant and natural bone could create stress shielding. This problem could be solved by creating special perforations in the surface of the titanium sample. This would decrease the effective cross-section and, consequently, the elastic modulus. However, sample strength could decrease as a result of using this technology. Therefore, topological optimization of the number, size, shape, and location of these perforations on the sample surface was required. A structural model of the titanium reinforcement with the optimal number, size, shape, and location of perforations was built in SOLIDWORKS Simulation 2018 SP3. The results of the mechanical-compression test on the solid-titanium samples were used to construct a material model in SOLIDWORKS Simulation 2018 SP3. The boundary conditions and loads were set; the model was under 250 MPa of uniaxial compression, a number corresponding to the maximal compressive strength of natural bone. In each iteration and during the calculations (topology optimization), the relative densities of the elements, calculated on the basis of the stress level of each element, were obtained. If the density value of the element were lower than the preset value, the element would be excluded from the finite-element model.

**Figure 3:**
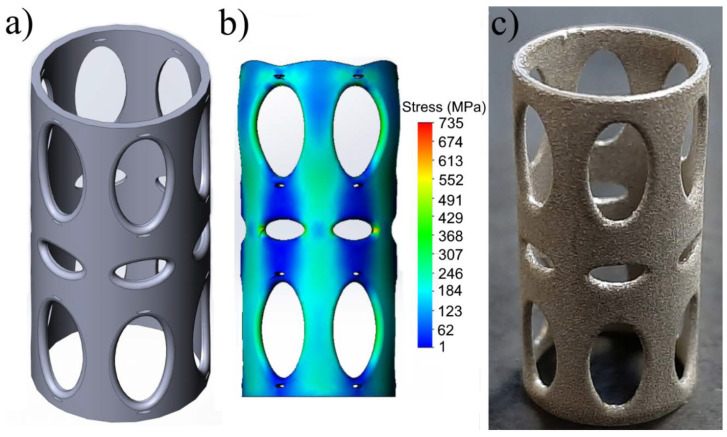
(**a**) Structural model of titanium reinforcement with reduced elastic modulus; (**b**) simulation of stress distribution in titanium-reinforcement structure with reduced elastic modulus under 250 MPa of stress; (**c**) photo of titanium sample with reduced elastic modulus prepared using three-dimensional (3D)-printing technology.

The developed model of the titanium-reinforcement structure with a reduced elastic modulus is shown in Figure 3a. The model consisted of 1.5 × 10^5^ elements and 7.8 × 10^5^ nodes. During the simulation, all of the nodes on the lower face of the cylinder were limited in all degrees of freedom. Convergence criteria were used for displacement and force. Tolerance was set at 1 × 10^−5^, and convergence-criteria values were set to 0.05 for both force and displacement. Stress distribution in the developed model at 250 MPa of compressive stress is shown in Figure 3b. According to the tests in SOLIDWORKS Simulation 2018 SP3, the titanium-reinforcement structure had an elastic modulus of about 30 GPa. Figure 3c shows a photo of a titanium-reinforcement sample with a reduced elastic modulus prepared using SLM technology.

### 2.4. UHMWPE/Titanium-Hybrid Implant Molding

GUR 1020 UHMWPE was used to prepare a porous scaffold according to the salt-leaching method described by Maksimkin et al. [15,33]. NaCl with a particle size ranging from 80 to 900 μm was used as soluble material. A UHMWPE/NaCl-composite powder was prepared through a solid-state mixing method in a planetary ball mill (Pulverisette 5, Fritsch, Idar-Oberstein, Germany) at low-energy conditions. UHMWPE and NaCl powder were mixed at a ratio of 1:9 by weight.

To create the UHMWPE/titanium-hybrid implant, it was necessary to form a porous UHMWPE scaffold inside the titanium-reinforcement samples. This porous UHMWPE layer needed to have the same characteristics as those of the porous UHMWPE scaffold described above; this was considered an important factor.

**Figure 4:**
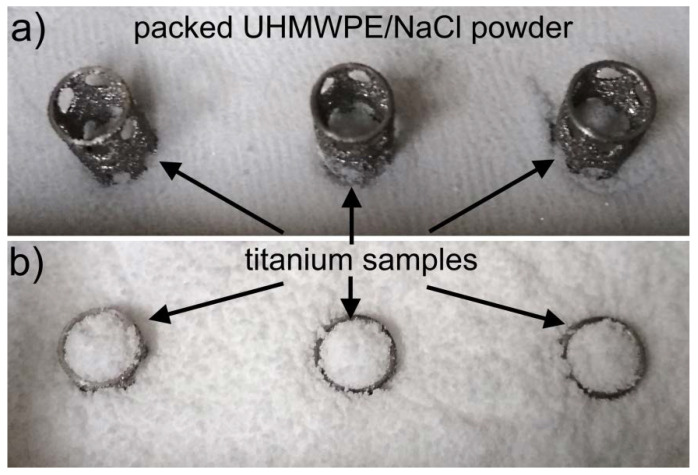
Photos showing loading process of UHMWPE/NaCl-composite powders and titanium-reinforcement samples in press mold. (**a**) Titanium-reinforcement samples placed vertically on to compacted UHMWPE/NaCl powder; (**b**) loading additional amount of UHMWPE/NaCl-composite powder into press mold.

A hot-pressing method was used to form the porous UHMWPE layer in the titanium-reinforcement samples. In the press mold, the UHMWPE/NaCl-composite powder and the titanium-reinforcement samples were loaded as follows. First, the UHMWPE/NaCl-composite powder was loaded into the press mold, and compacted at room temperature and 60 MPa of pressure, as this was necessary to prevent any deformation in the titanium-reinforcement samples. The thickness of the compacted UHMWPE/NaCl-composite layer was 5 mm. Second, the titanium-reinforcement samples were placed vertically onto the compacted UHMWPE/NaCl-composite layer, as shown in Figure 4a. Third, an additional amount of UHMWPE/NaCl-composite powder was loaded into the press mold, as shown in Figure 4b. The hot-pressing process was carried out at 180 °C and 40 MPa of pressure.

After the hot-pressing process, the plate consisting of the titanium-reinforcement samples and the UHMWPE/NaCl composites was taken out and washed in subcritical water at a temperature of 120 °C and pressure of 250 bar to remove the salt [34]. After removing the salt, the outermost porous UHMWPE layer around the titanium reinforcement was mechanically removed, so that the titanium-reinforcement samples filled with porous UHMWPE could be obtained. The adsorbed water in the UHMWPE pores was removed by drying at 70 °C for 3 h.

The thin solid layer on the surface of the hybrid implant was made from UHMWPE GUR 1020. UHMWPE cylinders with diameter of 15 mm, height of 22 m, and wall thickness of 0.5 mm were made using a hot-pressing process. UHMWPE cylinders were radially stretched to a diameter of 25 mm. Afterwards, the prepared titanium-reinforcement sample containing porous UHMWPE was put into the stretched solid-UHMWPE cylinder, and both were heated to a temperature of 100 °C. Because of the shape-memory effect in UHMWPE [35], the stretched solid-UHMWPE cylinder was tightly connected to the prepared titanium-reinforcement sample containing the porous UHMWPE. Thus, a thin solid layer on the surface of the hybrid implant was created.

## 3. Results and Discussion

**Figure 5:**
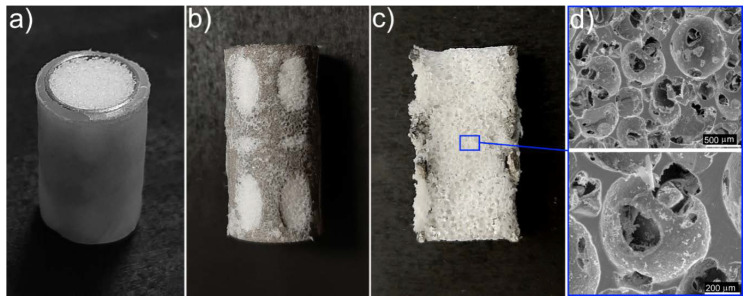
(**a**) Photo of prepared UHMWPE/titanium-hybrid implant; (**b**) titanium reinforcement with porous UHMWPE scaffold (thin solid UHMWPE layer was removed); (**c**) halved sample of titanium reinforcement containing porous UHMWPE scaffold; (**d**) SEM image of porous UHMWPE scaffold inside titanium reinforcement.

**Figure 6:**
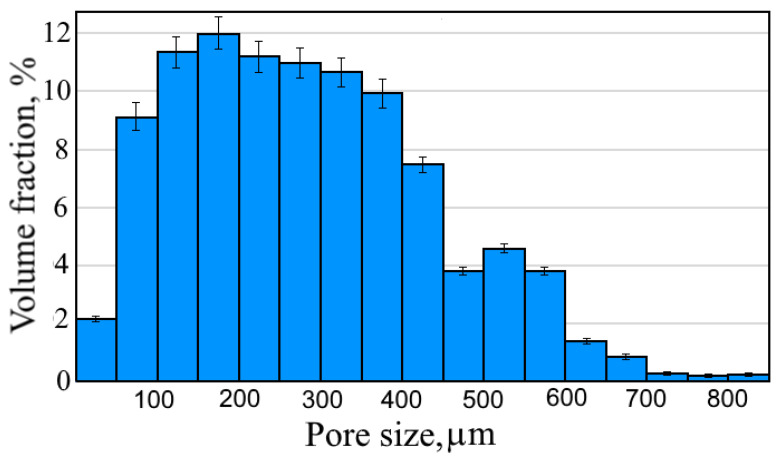
Pore-size distribution of porous UHMWPE scaffold inside titanium reinforcement.

Figure 5a shows a photo of the prepared UHMWPE/titanium-hybrid implant that consisted of a thin solid UHMWPE layer (giving it a smooth surface), titanium reinforcement (cortical-tissue simulation), and a porous UHMWPE scaffold (cancellous-tissue simulation). To investigate the porous UHMPWE scaffold’s structure, the thin solid UHMWPE layer was removed from the UHMWPE/titanium-hybrid implant (see Figure 5b), and the titanium reinforcement containing the porous-UHMWPE-scaffold sample was cut in half (see Figure 5c). The SEM image of this porous UHMWPE scaffold demonstrated that the pores were open and interconnected, as shown in Figure 5d. The initial individual UHMWPE particles were missing. All polymer particles were sintered. Figure 6 illustrates the distribution of pore sizes in the porous UHMWPE scaffold, which ranged from 50 to 850 μm. Average pore size was 250 μm. Volume porosity was 79 ± 2%. Adhesion between titanium and porous UHMWPE was of a physical nature because of the high surface roughness of the titanium sample.

The solid UHMWPE covering the UHMWPE/titanium-hybrid implant could be saturated with an antibiotic (e.g., amoxicillin) using supercritical-fluid-impregnation technologies, as illustrated in our previous article [36]. Adding an antibiotic to the solid UHMWPE could solve the problem of peri-implant inflammation that is often induced by opportunistic microflora.

Results of the mechanical-compression tests on the solid-titanium samples without perforations in their surface and on the hybrid implants are presented in Table 1. As a control reference, data on the compressive mechanical properties of natural bone were added to Table 1. The solid-titanium samples prepared by 3D printing had compressive strength of 835 ± 41 MPa, elastic modulus of 116 ± 4 GPa, and deformation of 1.1 ± 0.1%. The UHMWPE/titanium-hybrid implant had compressive strength of 256 ± 10 MPa, elastic modulus of 32.6 ± 4.5 GPa and deformation of 1.5 ± 0.1%. Simulated stress behavior of the titanium-reinforcement-model structure had good agreement with the obtained mechanical properties (see Figure 3b).

Obtained mechanical results demonstrated an effective decrease in the elastic modulus of the hybrid implant compared to that of the solid titanium because of the designed perforations in the surface of the hybrid implant’s titanium reinforcement, which the solid titanium did not have. The compressive mechanical properties of the UHMWPE/titanium-hybrid implants were very close to those of natural bone (see Table 1). Such mechanical behavior in the hybrid implants could minimize the occurrence of stress shielding in the bone.

**Figure 7:**
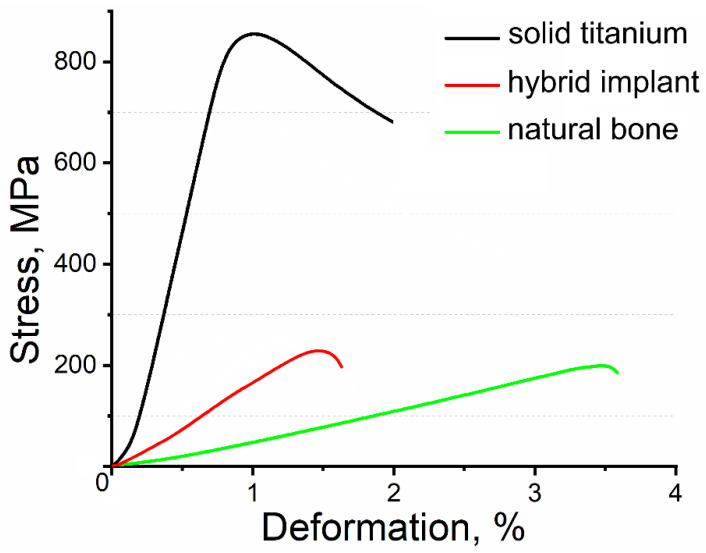
Stress-deformation diagram for solid titanium without perforations, UHMWPE/titanium-hybrid implant, and natural bone. Mechanical-compression tests on natural bone performed in similar conditions to those in place during tests on hybrid implants. Bone size corresponded to that of UHMWPE/titanium-hybrid implant. Canine elbow bone was used for bone-tissue samples.

Figure 7 displays the stress-deformation diagram for the solid titanium without perforations in its surface, the UHMWPE/titanium-hybrid implant and natural bone. The stress-deformation diagram shows differences in the elastic modulus and deformation behavior of the hybrid implant and natural bone. However, these differences were in the range of the bone properties’ statistical scatter, which depended on bone composition, porosity, age, gender, and functional demands.

## 4. Conclusions

In our previous work, porous UHMWPE with open and interconnected pores to provide the ideal environment for cell growth was prepared. However, the problem of the difference in the mechanical properties of synthetic implants and natural bone needed to be solved to successfully apply the developed scaffolds for replacing loaded areas of bone tissue. In this study, this problem was solved by developing a hybrid implant consisting of two main components: a porous UHMWPE scaffold that simulated cancellous tissue and titanium alloy Ti–6Al–4V that simulated cortical tissue in natural bone. An approach was proposed to reduce the elastic modulus of titanium by creating special perforations in the titanium-sample surface. These perforations could decrease the effective cross-section and, consequently, the elastic modulus. Topological optimization of the number, size, shape, and location of these perforations in the sample surface was performed using SOLIDWORKS Simulation.

Titanium samples were manufactured using selective-laser-melting technology. This technology allowed to manufacture specialized implants depending on the size of the bone-tissue area to be replaced.

A method for forming the porous UHMWPE scaffold inside the titanium reinforcement was proposed. SEM images of the porous UHMWPE scaffold demonstrated that pores were open and interconnected, and that all polymer particles were sintered. Pore size was in the range of 50–850 μm, average pore size was 250 μm, and volume porosity was 79 ± 2%. Adhesion between titanium and porous UHMWPE was of a physical nature because of the titanium sample’s high surface roughness. An examination of the obtained structure of the porous UHMWPE scaffold that formed inside the titanium reinforcement showed that this structure was similar to that of the porous scaffold described by Maksimkin et al. [15,33]. It could be suggested that the porous scaffold inside the titanium reinforcement had high osteogenic properties, as shown by the authors mentioned above [15].

Obtained mechanical results demonstrated an effective decrease in elastic modulus due to the designed perforations in the surface of the titanium reinforcement. The compressive mechanical properties of the UHMWPE/titanium-hybrid implants were very close to those of natural bone. Such mechanical behavior of the hybrid implants could minimize the occurrence of stress shielding in the bone.

**Figure 8:**
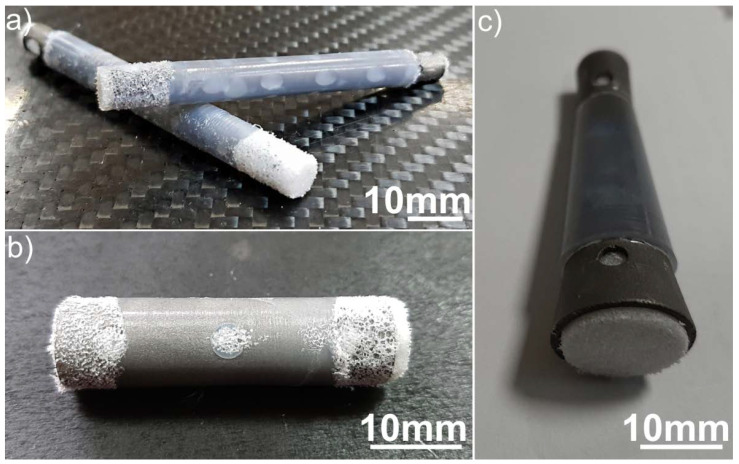
(**a**–**c**) Photos of prepared UHMWPE/titanium-hybrid implants used in real clinical cases.

The veterinary medical applications of the developed technology for producing UHMWPE/titanium-hybrid implants were tested in real clinical cases. Figure 8 shows photos of the obtained UHMWPE/titanium-hybrid implants used for bone-tissue replacement in a dog and two cats. These implants were prepared according to the size and shape of the replaced bone-tissue area; their mechanical properties were similar to those of the replaced bone. Surgical operations to place the implants were successful.

## Figures and Tables

**Table 1:** Mechanical properties of solid titanium, hybrid implant, and natural bone.

Material	Compressive Strength, MPa	Elastic Modulus, GPa	Deformation, %
Solid titanium without perforations	835 ± 41	116 ± 4	1.1 ± 0.1
Hybrid implant	256 ± 10	32.6 ± 4.5	1.2 ± 0.1
Natural bone [30,31,32]	130–230	7–30	1–6
